# Crystal structure of di­chlorido­{4-[(*E*)-(meth­oxy­imino-κ*N*)meth­yl]-1,3-thia­zol-2-amine-κ*N* ^3^}palladium(II)

**DOI:** 10.1107/S2056989014026619

**Published:** 2015-01-01

**Authors:** Viktorita V. Dyakonenko, Olga O. Zholob, Svitlana I. Orysyk, Vasily I. Pekhnyo

**Affiliations:** a State Scientific "Institution Institute for Single Crystals", National Academy of Science of Ukraine, 60 Lenina ave., Kharkiv 61001, Ukraine; b V.I. Vernadskii Institute of General and Inorganic Chemistry, National Academy of Sciences of Ukraine, 03680 Kyiv, Ukraine

**Keywords:** crystal structure, palladium, multi-functional ligand, 4-[(meth­oxy­imino)­meth­yl]-1,3-thia­zol-2-amine (MIMTA)

## Abstract

In the title compound, [PdCl_2_(C_5_H_7_N_3_OS)], the Pd^II^ atom adopts a distorted square-planar coordination sphere defined by two N atoms of the bidentate ligand and two Cl atoms. The mean deviation from the coordination plane is 0.029 Å. The methyl group is not coplanar with the plane of the metallacycle [torsion angle C—O—N—C = 20.2 (4)°]. Steric repulsion between the methyl group and atoms of the metallacycle is manifested by shortened intra­molecular H⋯C contacts of 2.27, 2.38 and 2.64 Å, as compared with the sum of the van der Waals radii of 2.87 Å. The amino group participates *via* one H atom in the formation of an intra­molecular N—H⋯Cl hydrogen bond. In the crystal, the other H atom of the amino group links mol­ecules *via* bifurcated N—H⋯(Cl,O) hydrogen bonds into chains parallel to [001].

## Related literature   

4-[(Meth­oxy­imino)­meth­yl]-1,3-thia­zol-2-amine (MIMTA) belongs to the class of polyfunctional oximes that are potential biologically active complexing agents (Dodoff *et al.*, 2009 ▸; Elo, 2004 ▸; Scaffidi-Domianello *et al.*, 2011 ▸; Donde & Patil, 2011 ▸; Kuwar *et al.*, 2006 ▸). Palladium complexes based on MIMTA are thus inter­esting in biomedicine (Orysyk *et al.*, 2013 ▸). For the structures of related complexes, see: Orysyk *et al.* (2015 ▸); Mokhir *et al.* (2002 ▸). For van der Waals radii, see: Zefirov (1997 ▸).
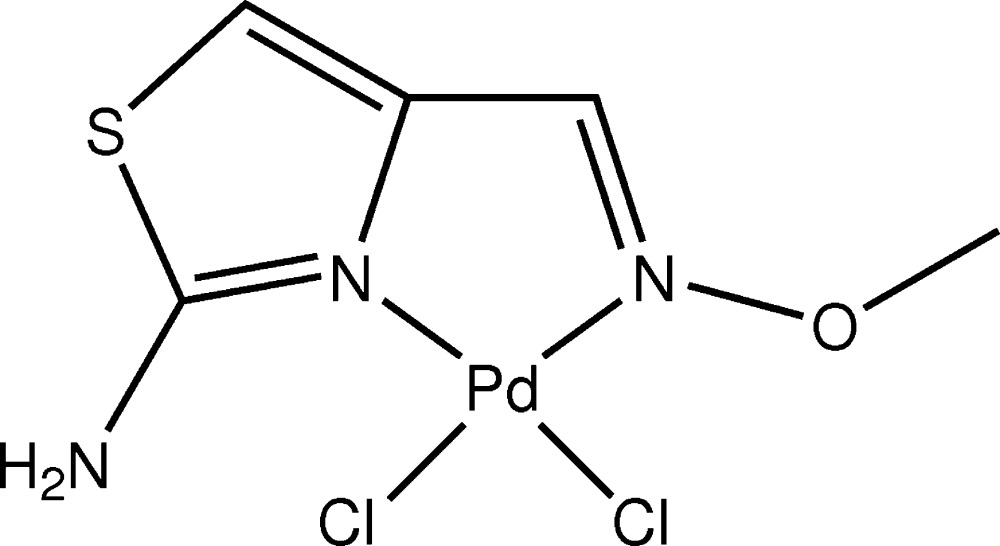


## Experimental   

### Crystal data  

[PdCl_2_(C_5_H_7_N_3_OS)]
*M* *_r_* = 334.50
Orthorhombic, 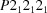

*a* = 4.347 (3) Å
*b* = 13.583 (2) Å
*c* = 16.411 (3) Å
*V* = 969.0 (7) Å^3^
*Z* = 4
Mo *K*α radiation
μ = 2.64 mm^−1^
*T* = 294 K
0.4 × 0.3 × 0.2 mm

### Data collection  

Agilent Xcalibur Sapphire3 diffractometer
Absorption correction: multi-scan (*CrysAlis RED*; Agilent, 2012 ▸) *T*_min_ = 0.742, *T*_max_ = 1.000
4284 measured reflections
2106 independent reflections
2028 reflections with *I* > 2σ(*I*)
*R*_int_ = 0.019

### Refinement  

*R*[*F* ^2^ > 2σ(*F* ^2^)] = 0.022
*wR*(*F* ^2^) = 0.047
*S* = 1.04
2106 reflections
120 parameters
H-atom parameters constrained
Δρ_max_ = 0.37 e Å^−3^
Δρ_min_ = −0.36 e Å^−3^
Absolute structure: Flack (1983), 969 Friedel pairs
Absolute structure parameter: 0.39 (4)

### 

Data collection: *CrysAlis CCD* (Agilent, 2012 ▸); cell refinement: *CrysAlis CCD*; data reduction: *CrysAlis RED* (Agilent, 2012 ▸); program(s) used to solve structure: *SHELXS97* (Sheldrick, 2008 ▸); program(s) used to refine structure: *SHELXL97* (Sheldrick, 2008 ▸); molecular graphics: *OLEX2* (Dolomanov *et al.*, 2009 ▸); software used to prepare material for publication: *OLEX2*.

## Supplementary Material

Crystal structure: contains datablock(s) I. DOI: 10.1107/S2056989014026619/wm5096sup1.cif

Structure factors: contains datablock(s) I. DOI: 10.1107/S2056989014026619/wm5096Isup2.hkl

**Fig. 1.**
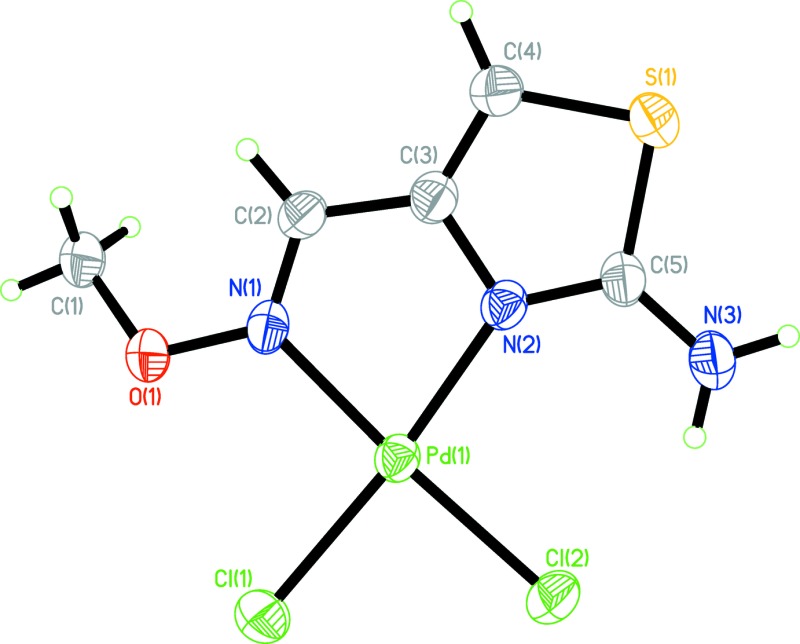
The molecular structure of the title compound. Displacement ellipsoids are drawn at the 50% probability level.

. DOI: 10.1107/S2056989014026619/wm5096fig1.tif
The mol­ecular structure of the title compound. Displacement ellipsoids are drawn at the 50% probability level.

**Fig. 2.**
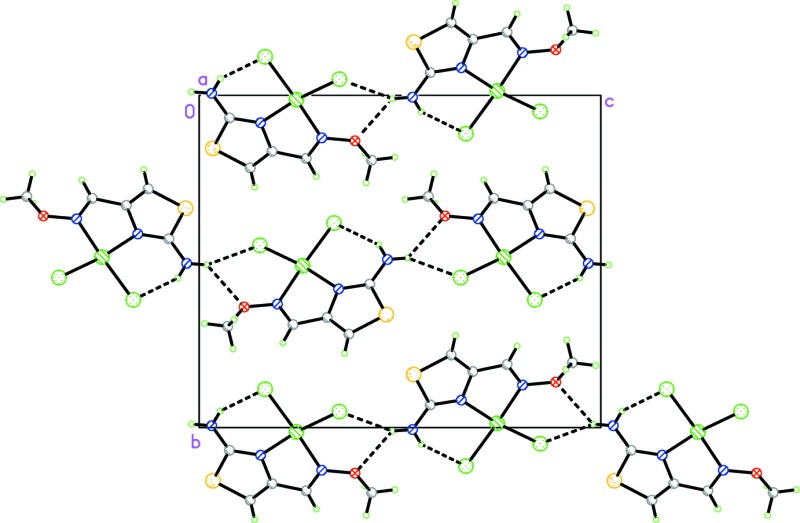
Crystal packing of the title compound with hydrogen bonds shown as dashed lines.

. DOI: 10.1107/S2056989014026619/wm5096fig2.tif
Crystal packing of the title compound with hydrogen bonds shown as dashed lines.

CCDC reference: 1037339

Additional supporting information:  crystallographic information; 3D view; checkCIF report

## Figures and Tables

**Table 1:** Hydrogen-bond geometry (, )

*D*H*A*	*D*H	H*A*	*D* *A*	*D*H*A*
N3H3*A*Cl2	0.86	2.34	3.124(3)	151
N3H3*B*Cl1^i^	0.86	2.48	3.280(3)	156
N3H3*B*O1^i^	0.86	2.45	3.015(3)	124

Symmetry code: (i) 
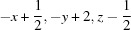
.

## supplementary crystallographic information

### S1. Experimental

PdCl_2_ (0.036 g, 0.2 mmol) was dissolved in 6*N* HCl (3 ml) at 313–323 K, and ethanol (7 ml) was added. To the resulting light-brown solution was added a hot solution of 4-[(methoxyimino)methyl]-1,3-thiazol-2-amine (0.031 g, 0.2 mmol), dissolved in ethanol (10 ml). The reaction mixture was stirred for one hour under reflux and cooled down to room temperature whereupon orange needle-like single crystals were filtered off, washed with ethanol and diethyl ether and dried in a vacuum desiccator over CaCl_2_. Yield: 0.045 g (65%).

### S2. Refinement

All hydrogen atoms were located from difference Fourier maps and constrained to ride on their parent atoms, with *U*_iso_ = 1.2*U*_eq_ (except *U*_iso_ = 1.5*U*_eq_ for the methyl group). The structure was refined from a crystal twinned by inversion (Flack parameter value 0.39 (4)).

### Crystal data

**Table:**

[PdCl_2_(C_5_H_7_N_3_OS)]	*D*_x_ = 2.293 Mg m^−^^3^
*M**_r_* = 334.50	Mo *K*α radiation, λ = 0.71073 Å
Orthorhombic, *P*2_1_2_1_2_1_	Cell parameters from 2494 reflections
*a* = 4.347 (3) Å	θ = 3.8–31.7°
*b* = 13.583 (2) Å	µ = 2.64 mm^−^^1^
*c* = 16.411 (3) Å	*T* = 294 K
*V* = 969.0 (7) Å^3^	, orange
*Z* = 4	0.4 × 0.3 × 0.2 mm
*F*(000) = 648	

### Data collection

**Table:**

Agilent Xcalibur Sapphire3 diffractometer	2106 independent reflections
Radiation source: Enhance (Mo) X-ray Source	2028 reflections with *I* > 2σ(*I*)
Graphite monochromator	*R*_int_ = 0.019
Detector resolution: 16.1827 pixels mm^-1^	θ_max_ = 27.5°, θ_min_ = 3.0°
ω scans	*h* = −5→5
Absorption correction: multi-scan (*CrysAlis RED*; Agilent, 2012)	*k* = −17→16
*T*_min_ = 0.742, *T*_max_ = 1.000	*l* = −21→21
4284 measured reflections	

### Refinement

**Table:**

Refinement on *F*^2^	Secondary atom site location: difference Fourier map
Least-squares matrix: full	Hydrogen site location: difference Fourier map
*R*[*F*^2^ > 2σ(*F*^2^)] = 0.022	H-atom parameters constrained
*wR*(*F*^2^) = 0.047	*w* = 1/[σ^2^(*F*_o_^2^) + (0.0225*P*)^2^ + 0.1807*P*] where *P* = (*F*_o_^2^ + 2*F*_c_^2^)/3
*S* = 1.04	(Δ/σ)_max_ = 0.002
2106 reflections	Δρ_max_ = 0.37 e Å^−^^3^
120 parameters	Δρ_min_ = −0.36 e Å^−^^3^
0 restraints	Absolute structure: Flack (1983), 969 Friedel pairs
Primary atom site location: structure-invariant direct methods	Absolute structure parameter: 0.39 (4)

### Special details

**Table:**

Geometry. All e.s.d.'s (except the e.s.d. in the dihedral angle between two l.s. planes) are estimated using the full covariance matrix. The cell e.s.d.'s are taken into account individually in the estimation of e.s.d.'s in distances, angles and torsion angles; correlations between e.s.d.'s in cell parameters are only used when they are defined by crystal symmetry. An approximate (isotropic) treatment of cell e.s.d.'s is used for estimating e.s.d.'s involving l.s. planes.
Refinement. Refinement of *F*^2^ against ALL reflections. The weighted *R*-factor *wR* and goodness of fit *S* are based on *F*^2^, conventional *R*-factors *R* are based on *F*, with *F* set to zero for negative *F*^2^. The threshold expression of *F*^2^ > σ(*F*^2^) is used only for calculating *R*-factors(gt) *etc*. and is not relevant to the choice of reflections for refinement. *R*-factors based on *F*^2^ are statistically about twice as large as those based on *F*, and *R*- factors based on ALL data will be even larger.

### Fractional atomic coordinates and isotropic or equivalent isotropic displacement parameters (Å^2^)

**Table:**

	*x*	*y*	*z*	*U*_iso_*/*U*_eq_	
Pd1	0.15497 (6)	0.986930 (16)	0.742144 (12)	0.02751 (7)	
Cl1	−0.1238 (2)	1.04846 (7)	0.84901 (5)	0.0396 (2)	
Cl2	−0.0192 (2)	1.11559 (6)	0.66418 (5)	0.0411 (2)	
S1	0.6968 (2)	0.84020 (7)	0.53414 (5)	0.0394 (2)	
O1	0.2794 (6)	0.86291 (19)	0.88793 (12)	0.0379 (6)	
N1	0.3271 (7)	0.87185 (19)	0.80514 (14)	0.0306 (6)	
N2	0.3996 (6)	0.91712 (19)	0.65287 (15)	0.0291 (6)	
N3	0.3636 (8)	1.0058 (2)	0.53113 (15)	0.0489 (8)	
H3A	0.2466	1.0498	0.5526	0.059*	
H3B	0.4153	1.0104	0.4807	0.059*	
C1	0.5024 (9)	0.8046 (3)	0.92925 (18)	0.0389 (8)	
H1A	0.4798	0.8132	0.9870	0.058*	
H1B	0.7050	0.8249	0.9130	0.058*	
H1C	0.4729	0.7366	0.9156	0.058*	
C2	0.4954 (8)	0.8100 (2)	0.76650 (18)	0.0328 (7)	
H2	0.5819	0.7552	0.7916	0.039*	
C3	0.5408 (8)	0.8314 (2)	0.68133 (18)	0.0315 (7)	
C4	0.7074 (9)	0.7815 (3)	0.62715 (19)	0.0375 (8)	
H4	0.8138	0.7236	0.6379	0.045*	
C5	0.4632 (8)	0.9318 (2)	0.57529 (18)	0.0317 (7)	

### Atomic displacement parameters (Å^2^)

**Table:**

	*U*^11^	*U*^22^	*U*^33^	*U*^12^	*U*^13^	*U*^23^
Pd1	0.03240 (12)	0.02459 (11)	0.02555 (10)	−0.00010 (10)	−0.00195 (9)	0.00015 (8)
Cl1	0.0450 (5)	0.0412 (5)	0.0327 (4)	0.0059 (4)	0.0021 (4)	−0.0055 (3)
Cl2	0.0511 (5)	0.0326 (4)	0.0394 (4)	0.0092 (4)	−0.0021 (4)	0.0062 (4)
S1	0.0506 (6)	0.0390 (5)	0.0286 (4)	0.0037 (5)	0.0042 (4)	−0.0028 (3)
O1	0.0456 (14)	0.0444 (14)	0.0237 (9)	0.0096 (12)	0.0026 (10)	0.0059 (9)
N1	0.0373 (15)	0.0304 (14)	0.0240 (11)	−0.0015 (15)	−0.0013 (12)	0.0048 (10)
N2	0.0361 (16)	0.0239 (13)	0.0273 (12)	−0.0007 (12)	−0.0025 (11)	0.0012 (10)
N3	0.077 (2)	0.0424 (17)	0.0276 (12)	0.014 (2)	0.0087 (14)	0.0054 (12)
C1	0.045 (2)	0.045 (2)	0.0260 (14)	0.006 (2)	−0.0007 (16)	0.0053 (14)
C2	0.0409 (18)	0.0273 (15)	0.0303 (15)	0.0048 (15)	−0.0018 (15)	0.0024 (13)
C3	0.0388 (18)	0.0277 (17)	0.0282 (14)	−0.0013 (15)	−0.0032 (14)	−0.0001 (13)
C4	0.045 (2)	0.0342 (18)	0.0333 (15)	0.0069 (17)	−0.0021 (16)	−0.0014 (13)
C5	0.0385 (18)	0.0316 (18)	0.0251 (14)	−0.0029 (16)	0.0012 (14)	0.0012 (13)

### Geometric parameters (Å, º)

**Table:**

Pd1—Cl1	2.2897 (10)	N3—H3A	0.8600
Pd1—Cl2	2.2943 (9)	N3—H3B	0.8600
Pd1—N1	2.018 (3)	N3—C5	1.313 (4)
Pd1—N2	2.044 (3)	C1—H1A	0.9600
S1—C4	1.723 (3)	C1—H1B	0.9600
S1—C5	1.742 (3)	C1—H1C	0.9600
O1—N1	1.380 (3)	C2—H2	0.9300
O1—C1	1.423 (4)	C2—C3	1.441 (4)
N1—C2	1.282 (4)	C3—C4	1.332 (5)
N2—C3	1.397 (4)	C4—H4	0.9300
N2—C5	1.318 (4)		
			
Cl1—Pd1—Cl2	88.54 (4)	O1—C1—H1B	109.5
N1—Pd1—Cl1	94.96 (8)	O1—C1—H1C	109.5
N1—Pd1—Cl2	176.43 (8)	H1A—C1—H1B	109.5
N1—Pd1—N2	79.34 (10)	H1A—C1—H1C	109.5
N2—Pd1—Cl1	173.65 (8)	H1B—C1—H1C	109.5
N2—Pd1—Cl2	97.20 (8)	N1—C2—H2	122.4
C4—S1—C5	90.16 (16)	N1—C2—C3	115.2 (3)
N1—O1—C1	114.6 (2)	C3—C2—H2	122.4
O1—N1—Pd1	121.1 (2)	N2—C3—C2	115.6 (3)
C2—N1—Pd1	117.8 (2)	C4—C3—N2	116.1 (3)
C2—N1—O1	121.0 (3)	C4—C3—C2	128.3 (3)
C3—N2—Pd1	112.1 (2)	S1—C4—H4	125.0
C5—N2—Pd1	137.0 (2)	C3—C4—S1	110.0 (3)
C5—N2—C3	110.9 (3)	C3—C4—H4	125.0
H3A—N3—H3B	120.0	N2—C5—S1	112.9 (2)
C5—N3—H3A	120.0	N3—C5—S1	121.7 (2)
C5—N3—H3B	120.0	N3—C5—N2	125.4 (3)
O1—C1—H1A	109.5		
			
Pd1—N1—C2—C3	0.1 (4)	N1—C2—C3—N2	0.9 (5)
Pd1—N2—C3—C2	−1.4 (4)	N1—C2—C3—C4	178.7 (4)
Pd1—N2—C3—C4	−179.5 (3)	N2—Pd1—N1—O1	175.8 (3)
Pd1—N2—C5—S1	179.58 (19)	N2—Pd1—N1—C2	−0.7 (3)
Pd1—N2—C5—N3	−1.1 (6)	N2—C3—C4—S1	−0.2 (4)
Cl1—Pd1—N1—O1	−7.0 (2)	C1—O1—N1—Pd1	−156.2 (2)
Cl1—Pd1—N1—C2	176.4 (3)	C1—O1—N1—C2	20.2 (4)
Cl1—Pd1—N2—C3	−25.3 (9)	C2—C3—C4—S1	−178.0 (3)
Cl1—Pd1—N2—C5	155.6 (5)	C3—N2—C5—S1	0.5 (4)
Cl2—Pd1—N1—O1	161.6 (12)	C3—N2—C5—N3	179.8 (3)
Cl2—Pd1—N1—C2	−14.9 (15)	C4—S1—C5—N2	−0.5 (3)
Cl2—Pd1—N2—C3	−179.7 (2)	C4—S1—C5—N3	−179.8 (3)
Cl2—Pd1—N2—C5	1.2 (3)	C5—S1—C4—C3	0.4 (3)
O1—N1—C2—C3	−176.4 (3)	C5—N2—C3—C2	177.9 (3)
N1—Pd1—N2—C3	1.1 (2)	C5—N2—C3—C4	−0.2 (4)
N1—Pd1—N2—C5	−178.0 (4)		

### Hydrogen-bond geometry (Å, º)

**Table:**

*D*—H···*A*	*D*—H	H···*A*	*D*···*A*	*D*—H···*A*
N3—H3*A*···Cl2	0.86	2.34	3.124 (3)	151
N3—H3*B*···Cl1^i^	0.86	2.48	3.280 (3)	156
N3—H3*B*···O1^i^	0.86	2.45	3.015 (3)	124

Symmetry code: (i) −*x*+1/2, −*y*+2, *z*−1/2.
