# Supplementary figures and images for: Crystal structure of di­chlorido­{4-[(E)-(meth­oxy­imino-κN)meth­yl]-1,3-thia­zol-2-amine-κN 3}palladium(II)
Source: Acta Crystallogr E Crystallogr Commun. 2015 Jan 1;71(Pt 1):m10–1. doi: 10.1107/S2056989014026619 (PMC4331851; doi:10.1107/S2056989014026619)

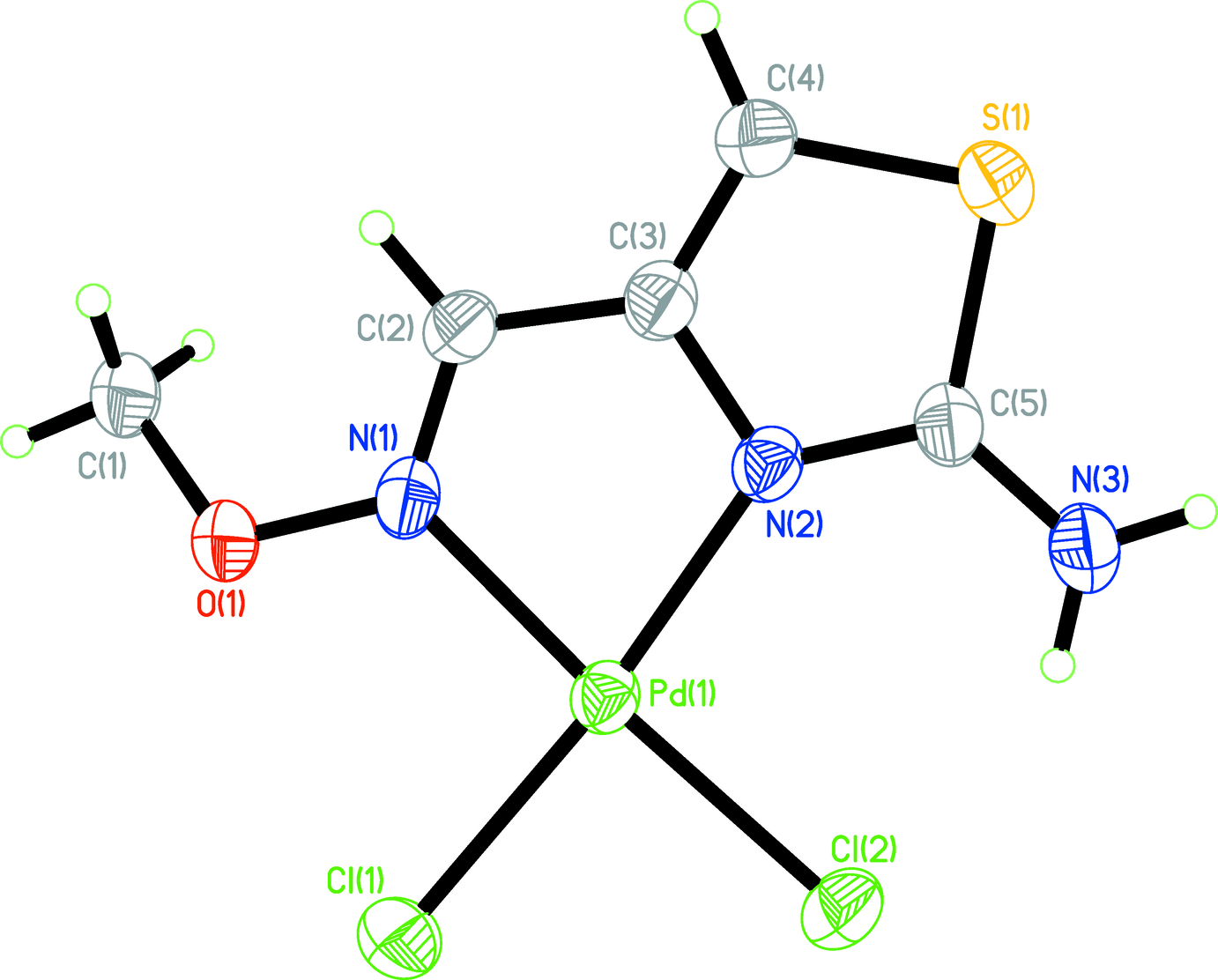

Supplement: Supplementary file 3 [file e-71-00m10-fig1.tif]

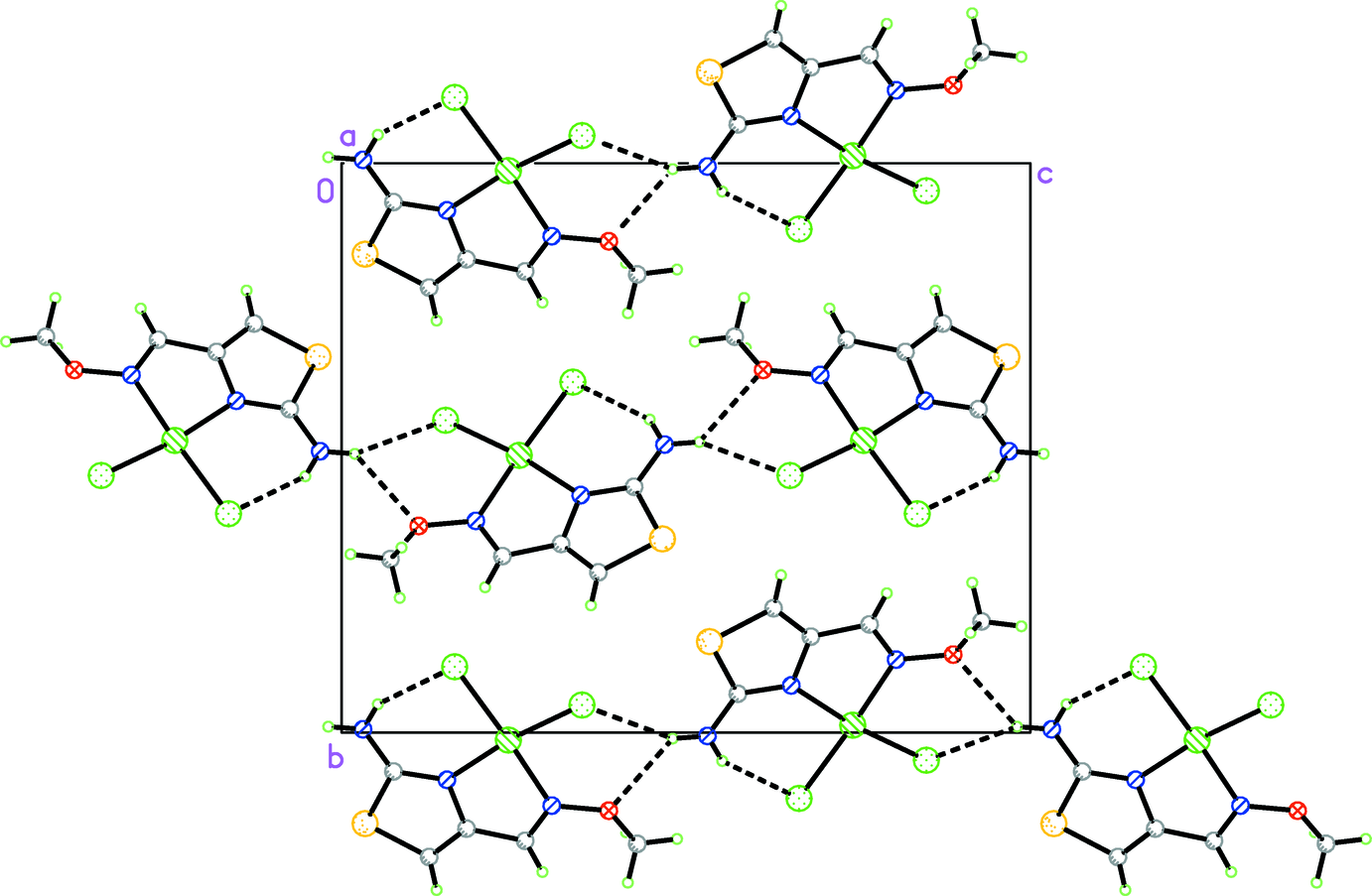

Supplement: Supplementary file 4 [file e-71-00m10-fig2.tif]
